# The prevalence of human papillomavirus infections and associated risk factors in men-who-have-sex-with-men in Cape Town, South Africa

**DOI:** 10.1186/s12879-016-1706-9

**Published:** 2016-08-22

**Authors:** Etienne E. Müller, Kevin Rebe, Tobias F. Chirwa, Helen Struthers, James McIntyre, David A. Lewis

**Affiliations:** 1 Centre for HIV and Sexually Transmitted Infections, National Institute for Communicable Diseases, National Health Laboratory Service, Johannesburg, South Africa; 2 Anova Health Institute, Johannesburg, South Africa; 3 Anova Health Institute, Cape Town, South Africa; 4 Department of Medicine, Division of Infectious Diseases and HIV Medicine, University of Cape Town, Cape Town, South Africa; 5 Division of Epidemiology and Biostatistics, School of Public Health, University of the Witwatersrand, Johannesburg, South Africa; 6 Division of Epidemiology & Biostatistics, School of Public & Family Medicine, University of Cape Town, Cape Town, South Africa; 7 Western Sydney Sexual Health Centre, Western Sydney Local Health District, Parramatta, Australia; 8 Marie Bashir Institute for Infectious Diseases and Biosecurity & Sydney Medical School-Westmead, University of Sydney, Sydney, Australia

**Keywords:** HPV, Anal, Oro-pharyngeal, Urine, MSM, Risk factors, South Africa

## Abstract

**Background:**

We investigated the prevalence of human papillomavirus (HPV) infection and associated behavioural risk factors in men-who-have-sex-with-men (MSM) attending a clinical service in Cape Town, South Africa.

**Methods:**

MSM were enrolled at the Ivan Toms Centre for Men’s Health in Cape Town. A psychosocial and sexual behavioral risk questionnaire was completed for each participant and urine, oro-pharyngeal and anal swabs were collected for HPV testing using the Linear Array HPV Genotyping Test. Logistic regression analyses were performed to determine sexual risk factors associated with HPV infection at the three anatomical sites.

**Results:**

The median age of all 200 participants was 32 years (IQR 26-39.5), of which 31.0 % were black, 31.5 % mixed race/coloured and 35.5 % white. The majority of the participants (73.0 %) had completed high school, 42.0 % had a tertiary level qualification and 69.0 % were employed. HPV genotypes were detected in 72.8 % [95 % CI: 65.9–79.0 %], 11.5 % [95 % CI: 7.4–16.8 %] and 15.3 % [95 % CI: 10.5–21.2 %] of anal, oro-pharyngeal and urine specimens, respectively. Prevalence of high-risk (HR)-HPV types was 57.6 % [95 % CI: 50.3–64.7 %] in anal samples, 7.5 % [95 % CI: 4.3–12.1 %] in oro-pharyngeal samples and 7.9 % [95 % CI: 4.5–12.7 %] in urine, with HPV-16 being the most common HR-HPV type detected at all sites. HPV-6/11/16/18 was detected in 40.3 % [95 % CI: 33.3–47.6 %], 4.5 % [95 % CI: 2.1–8.4 %] and 3.2 % [95 % CI: 1.2–6.8 %] of anal, oro-pharyngeal and urine samples, respectively. Multiple HPV types were more common in the anal canal of MSM while single HPV types constituted the majority of HPV infections in the oropharynx and urine. Among the 88 MSM (44.0 %) that were HIV positive, 91.8 % [95 % CI: 83.8–96.6 %] had an anal HPV infection, 81.2 % [95 % CI: 71.2–88.8 %] had anal HR-HPV and 85.9 % [95 % CI: 76.6–92.5 %] had multiple anal HPV types. Having sex with men only, engaging in group sex in lifetime, living with HIV and practising receptive anal intercourse were the only factors independently associated with having any anal HPV infection.

**Conclusions:**

Anal HPV infections were common among MSM in Cape Town with the highest HPV burden among HIV co-infected MSM, men who have sex with men only and those that practiced receptive anal intercourse. Behavioural intervention strategies and the possible roll-out of HPV vaccines among all boys are urgently needed to address the high prevalence of HPV and HIV co-infections among MSM in South Africa.

## Background

Human papillomavirus (HPV) is the most common sexually transmitted viral infection worldwide and is associated with occurrence of warts (condylomas) and a variety of cancers in both men and women [1]. The outcome of HPV infection depends on the specific HPV type/s present and can range from asymptomatic infection to severe squamous cell malignancies. Low-risk HPV types (such as types 6 and 11) are associated with anogenital warts and mild dysplasia, while high-risk types (such as 16 and 18) are associated with high-grade dysplasia and cancers of the cervix, vulva, vagina, urethra, penis, anus and oropharynx [1]. HPV is an independent risk factor for the acquisition of HIV [2]. HIV infection is also associated with an increased risk of acquiring new HPV infections and a reduction in the rate of HPV clearance [3]. South Africa has one of the highest HIV prevalence rates globally with an estimated prevalence of 12.2 % in the general population in 2012 [4]. HIV is especially prevalent among men-who-have-sex-with-men (MSM), with estimated prevalence rates of 22.3 %, 26.8 % and 48.2 % in Cape Town, Johannesburg and Durban, respectively [5]. Unprotected receptive anal intercourse is up to sixteen times more likely to transmit HIV than unprotected vaginal sex, placing MSM at higher risk of acquiring and transmitting HIV [6]. A meta-analysis of 53 studies reported HPV co-infection in 89 % - 93 % of HIV-infected MSM [7].

HPV infection in men is an important concern due to its association with anal, urethral, penile and oro-pharyngeal cancers. In South Africa, relatively little is known about the incidence rates of these cancers in men, and although these cancers are less common than cervical cancer, their association with HPV infection make them amenable to similar preventative measures as those for cervical cancer. The risk of developing invasive anal cancer was 17 times higher among MSM than in men-who-have-sex-with-women (MSW) and the risk doubled in HIV-infected MSM [8]. A number of studies reported anal HPV prevalence rates of 30–70 % in HIV-negative MSM and 71–100 % in HIV-positive MSM [9–12]. Oral HPV infection is less common in adults than anogenital HPV infection but is still strongly associated with HIV infection, high-risk sexual practices, especially engaging in oro-genital sex and having a high number of sexual partners [13, 14]. HPV can also be found in the urinary tract, indicating HPV infection in either the distal or proximal urethra, prostate and/or urinary bladder [15]. There are currently three HPV vaccines available that were designed to prevent HPV-associated cancer to certain HR-HPV genotypes. These HPV vaccines include the bivalent Cervarix® HPV vaccine (GSK Aspen) (targeting HPV types 16 and 18), the quadrivalent Gardasil® HPV vaccine (MSD) (targeting HPV types 6, 11, 16 and 18) as well as the 9-valent Gardasil® 9 HPV vaccine (MSD) (targeting HPV types 6, 11, 16, 18, 31, 33, 45, 52 and 58).

Epidemiological research has remained very limited among the MSM population in South Africa and as a result there are currently no HPV prevalence data for MSM. Due to high HIV prevalence rates among MSM in South Africa, this group has been identified by the South African Department of Health for targeted healthcare interventions and HIV prevention programs [16]. This study will therefore aim to investigate the prevalence of HPV genotypes at different anatomical sites (anus, oropharynx and urine) in a sample of 200 MSM seeking care at a dedicated men’s clinic in Cape Town, South Africa.

## Methods

### Population and sample selection

This study is a secondary analysis of data collected during a cross-sectional study of symptomatic and asymptomatic STIs among MSM in Cape Town, South Africa [17]. A convenience sample of 200 sequential MSM were enrolled by Health4Men (Anova Health Institute) at the Ivan Toms Centre for Men’s Health (ITCMH) in Cape Town during 2011 and 2012. These MSM were mostly asymptomatic (71.0 %) for STIs and attended the ITCMH for HIV, sexual health and/or mental health care and for commodities such as condoms and lubricants. The inclusion criteria for study participation included being 18 years of age or older, having sex with other men within one year prior to recruitment, understanding the study procedures, risks and benefits and willingness to give informed consent. Study participants attended two study-related visits two weeks apart. During the first visit participants were assessed for STI symptoms and examined for STI and HIV-related clinical conditions. All participants completed a researcher administered study questionnaire and provided a self-collected urine specimen and clinician collected rectal and oro-pharyngeal swab. These specimens were tested for *Neisseria gonorrhoeae* and *Chlamydia trachomatis* using the RNA-based Aptima Combo 2 assay (Hologic-Gen-Probe, San Diego, CA, USA.) and stored at −70 °C for HPV testing. At the follow-up visit, results were relayed to all participants and diagnosed conditions were treated according to the South African Department of Health’s first-line comprehensive management and control of STIs protocol [18]. All 200 participants gave permission for their specimens to be used for future research. Ethical approvals for this study were obtained from the Human Research Ethics Committee (Medical) of the University of the Witwatersrand [protocol M120454] and the Bioethics Department at the University of Cape Town [protocol 419/2011].

### Psychosocial and sexual behavioural data collection

Socio-demographic and sexual behavior characteristics were collected by designated study staff for the primary study through personal interviews using a standardized questionnaire. Circumcision status was confirmed during clinical examination. A sexual partner matrix (SPM) for the last 10 sexual encounters in the past 12 months (or less if the participant had less than 10 sexual encounters in the past 12 months) was completed for each participant. The SPM recorded the gender of each partner and condom use during sexual practices that included receptive and insertive anal intercourse, oral sex and oro-anal sex. Vaginal intercourse was recorded for men who had sexual encounters with female partners in the past 12 months.

### HPV detection and genotyping

HPV testing was performed at the STI Section, Centre for HIV and STIs, National Institute for Communicable Diseases (NICD), in Johannesburg. Three archived specimens for each of the 200 enrolled MSM participants were analysed, which included a first-void urine specimen, an oro-pharyngeal swab and an anal swab, which were all previously stored at −70 °C at the STI Section, Centre for HIV and STIs, NICD.

Genomic DNA was extracted from urine and swab specimens using the MagNAPure Compact Automated DNA Extractor (Roche) and used for HPV genotyping. The Linear Array (LA) HPV Genotyping Test (Roche Molecular Systems, Inc., Branchburg, NJ, USA) was used to determine the HPV genotype distribution in these 600 specimens. The LA test amplified the target HPV DNA for 37 anogenital HPV genotypes, which included 24 low risk HPV types (6, 11, 26, 40, 42, 53, 54, 55, 61, 62, 64, 66, 67, 69, 70, 71, 72, 73, 81, 82, 83, 84, IS39 and CP6108) and 13 high risk HPV types (16, 18, 31, 33, 35, 39, 45, 51, 52, 56, 58, 59 and 68). HPV-52 was only recorded positive in the absence of HPV types 33, 35 and 58 due to the combined probe used for these 4 types in the LA assay. The β-globin gene was amplified as a control for cell adequacy, extraction and amplification. Samples with a negative β-globin result and a positive HPV DNA result were considered valid and adequate for analyses.

### Detection of HIV and other STIs

Laboratory detection of HIV, syphilis, gonorrhea and chlamydia infection at the urethral, anal and oro-pharyngeal sites were previously determined for the primary study [17]. Syphilis testing was done on site using the SD Bioline rapid syphilis test and HIV serostatus was determined using both the SD Bioline HIV 1/2 3.0 test (Standard Diagnostics, Korea) and the Alere Determine HIV-1/2 kit (Alere Medical, Japan). Specimens were previously analysed for gonorrheal and chlamydial infection at the STI Section, NICD, using the Aptima 2 Combo assay (Hologic-Gen-Probe, San Diego, CA, USA.).

### Data confidentiality

Individual specimens and questionnaire data were delinked from all personal identifiers and each participant was assigned with a unique random HPV study number to ensure anonymity. There were no documents in existence linking original study numbers with HPV study numbers. The HPV genotyping results obtained were therefore not relayed to any patients and the results were only used for research purposes.

### Variables and definitions

The main outcome variables consisted of type-specific HPV prevalence, any HPV type, HR-HPV types and multiple HPV types. A participant was considered to have a HR-HPV infection if he tested positive for one or more HR- HPV genotypes, irrespective of the participant being co-infected with one or more LR-HPV genotypes. Multiple HPV was defined in participants with two or more HR or LR HPV genotypes. Socio-demographic variables of interest included age, gender, ethnicity, dwelling location, level of education, job status and income. For the purpose of this study MSM consisted of MSM not having sex with women (MSM only) and men who had sex with both men and women (MSMW). Additional sexual behaviours included last sex with men and women, number of male and female partners in the past 12 months, having a main partner, sex at sex-on-site venues in past 12 months, sex with a partner met on the internet in past 12 months, engaging in group sex in past 12 months and lifetime, sharing sex toys, participating in transactional sex, sex while under the influence of alcohol and drugs, lubrication used during sex and having an STI diagnosed in the past 12 months.

### Statistical analyses

HPV prevalence and questionnaire data were entered and analysed using both Epi Info version 7 (USDHHS, Centers for Disease Control and Prevention) and STATA SE version 14.0 (Stata Corporation, College Station, Texas, USA). Descriptive statistics included frequency tables for categorical variables such as level of education, HR-HPV and multiple HPV and summary measures i.e. mean and standard deviation (SD) or median and inter-quartile range (IQR) for continuous variables such as age. Factors associated with HPV infection, comparing differences between MSM only and MSMW and differences between HIV-positive and HIV-negative participants were assessed with Pearson chi-square test and Fisher’s exact test for categorical variables and Student’s *t*-test for continuous variables. Factors with a *p*-value of ≤0.05 in the univariate logistic regression analyses were included in the multiple logistic regression model to determine possible associations with outcome variables. The HPV outcome variables included HPV prevalence rates in this selected study population for any HPV, HR-HPV and multiple HPV at the 3 anatomical sites and their 95 % confidence intervals (CI); crude odds ratios (COR) and adjusted odds ratios (AOR) and its 95 % CI were calculated.

## Results

### Demographic and behavioural characteristics

Baseline characteristics, sexual behaviour and STI risks for the 200 participants have been published [17]. Briefly, the median age of all 200 participants was 32 years (IQR 26–39.5) with three-quarters (150) under 40 years of age. A total of fifteen participants (7.5 %) identified as transgender women (TGW; male to female). All three major South African racial groups were equally represented with 62 (31.0 %) black, 63 (31.5 %) mixed race/coloured and 71 (35.5 %) white participants. Baseline and sexual behavioural characteristics of men who have sex with men only (MSM only) and men who have sex with men and women (MSMW) are summarised in Table 1.

**Table 1:** Baseline and sexual behavioural characteristics stratified according to men who have sex with men only (MSM only) and men who have sex with men and women (MSMW)

Variable	No. (%) of men			*p*-value
	MSM only (*n* = 155)	MSMW (*n* = 45)	Totals (*n* = 200)	
Age [years(IQR)]	33 (26–40)	28 (24–37)	32 (26–39.5)	0.025
Gender				
Male	140 (90.3)	45 (100.0)	185 (92.5)	0.030
Transgender	15 (9.7)	0 (0.0)	15 (7.5)	
Ethnicity				
Black	48 (31.0)	14 (31.1)	62 (31.0)	˂0.001
Coloured	38 (24.5)	25 (55.6)	63 (31.5)	
White	65 (41.9)	6 (13.3)	71 (35.5)	
Other	4 (2.6)	0 (0.0)	4 (2.0)	
Education				
Completed high school	122 (78.7)	33 (73.3)	145 (72.5)	˂0.001
Tertiary qualification	73 (47.1)	11 (24.4)	84 (42.0)	
Transactional sex				
No	112 (72.3)	11 (24.4)	123 (61.5)	˂0.001
Yes, paid money for sex	17 (11.0)	7 (15.6)	24 (12.0)	
Yes, received money for sex	21 (13.5)	15 (33.3)	36 (18.0)	
Yes, paid and received money for sex	5 (3.2)	12 (26.7)	17 (8.5)	
Sex while under the influence of drugs				
Yes	73 (47.1)	32 (71.1)	105 (52.5)	0.004
No	82 (52.9)	13 (28.9)	95 (47.5)	
HIV serostatus				
Positive	81 (52.3)	7 (15.6)	88 (44.0)	˂0.001
Negative	74 (47.7)	38 (84.4)	112 (56.0)	
Frequency of insertive anal sex				
Yes	104 (67.1)	39 (86.7)	143 (71.5)	0.010
No	51 (32.9)	6 (13.3)	57 (28.5)	
Frequency of receptive anal sex				
Yes	125 (80.6)	15 (33.3)	140 (70.0)	˂0.001
No	30 (19.4)	30 (66.7)	60 (30.0)	
Frequency of receptive oral sex				
Yes	148 (95.5)	26 (57.8)	174 (87.0)	˂0.001
No	7 (4.5)	19 (42.2)	26 (13.0)	
Performing oro-anal sex (rimming)				
Yes	67 (43.2)	10 (22.2)	77 (38.9)	0.011
No	88 (56.8)	35 (77.8)	123 (61.5)	

The majority of MSM participants (155; 77.5 %) indicated that they had sex with men only while 45 (22.5 %) had sex with both men and women in the preceding 12 months. More than a third of all participants (77; 38.5 %), reported transactional sex (paid and/or received money for sex) in the past year. Significantly more MSMW reported participating in transactional sex in the past year (*p* < 0.001) and having had sex while under the influence of drugs in their lifetime (*p* = 0.004). More than half (52.3 %) of all MSM only participants were HIV positive while only 15.6 % of MSMW had an HIV infection (*p* < 0.001).

Insertive and receptive anal sex in the last 12 months were reported by 143 (71.5 %) and 140 (70.0 %) participants, respectively. Among MSMW, insertive anal sex was more commonly practised than receptive anal sex (86.5 % vs 33.3 %), while MSM only reported more receptive anal sex than insertive anal sex (80.6 % vs 67.1 %). Compared to MSM only, significantly more MSMW practised insertive anal sex (*p* = 0.010) and less receptive anal sex (*p* < 0.001). MSM only participants practised significantly more receptive oral sex (95.5 % vs 57.8 %) and insertive oro-anal sex (43.2 % vs 22.2 %) in the last 12 months. The SPM analysis revealed that 36 (80.0 %) MSMW participants had a female partner among their last 10 sexual partners. Performing vaginal intercourse was reported by 34 (94.4 %) of these MSMW participants, performing anal sex on women was less common (15/36; 41.7 %), while performing and receiving oral sex with women was reported by 29 (80.6 %) and 22 (61.1 %) participants, respectively. Performing and receiving oro-anal sex with women was only reported by 7/36 (19.4 %) participants.

### HIV and STI prevalence

As previously reported, 47 (23.5 %) participants reported having an STI diagnosis within the previous year, mostly with a single STI episode (80.9 % of cases) [17]. Eighty eight (44.0 %) participants were HIV positive with the majority (74.1 %) having CD4 counts below 500 cells/μl. Twenty two (11.0 %) participants tested positive for syphilis antibodies. *Neisseria gonorrhoeae* and *Chlamydia trachomatis* infections were detected in 32 (16 %) and 23 (11.5 %) MSM, respectively.

### HPV prevalence and genotype distribution

**Fig. 1:**
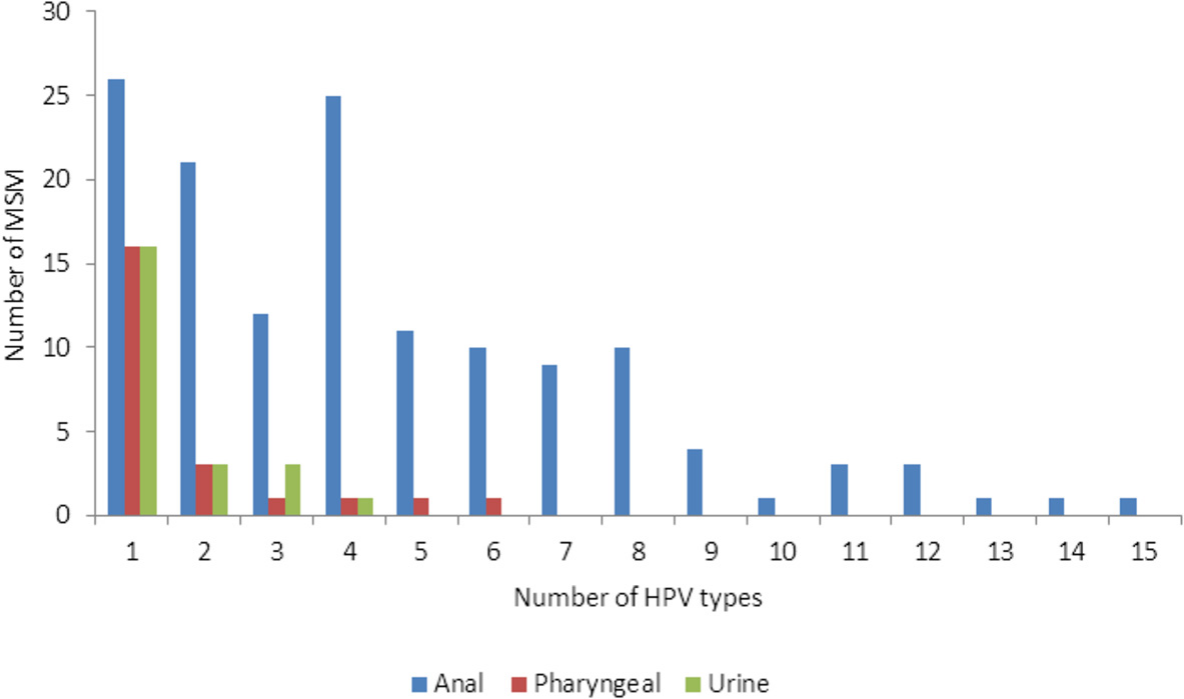
Number of HPV types in anal, oro-pharyngeal and urine specimens obtained from MSM participants in Cape Town

As shown in Tables 2 and 3, a total of 191 anal swabs (95.5 %), 200 oro-pharyngeal swabs (100 %) and 190 urines (95 %) had a valid HPV DNA result and were ncluded in the analysis; test strips lacking human beta globin DNA detection were reported as invalid. An overall HPV prevalence of 75 % (150/200) [95 % CI: 68.4–80.8 %] was observed for all patients, irrespective of the site of HPV positivity. HPV genotypes were detected in 139/191 (72.8 %) [95 % CI: 65.9–79.0 %], 23/200 (11.5 %) [95 % CI: 7.4–16.8 %] and 29/190 (15.3 %) [95 % CI: 10.5–21.2 %] of anal, oro-pharyngeal and urine specimens, respectively. The mean number of HPV types observed in anal, oro-pharyngeal and urine specimens were 4.47, 1.74 and 1.41 HPV types in HPV-positive MSM, respectively. Multiple HPV types were observed in 113/191 (59.2 %) [95 % CI: 51.8–66.2 %] of anal specimens, 7/200 (3.5 %) [95 % CI: 1.4–7.1 %] of oro-pharyngeal specimens and 7/190 (3.7 %) [95 % CI: 1.5–7.4 %] of urine specimens (Fig. 1). The most HPV types observed in an anal specimen was 15, followed by 6 HPV types in an oro-pharyngeal specimen and 4 HPV types in a urine specimen.

**Table 2:** HPV and HIV-co-infection at 3 anatomical sites among MSM in Cape Town, South Africa

HPV type classification	ANAL (*n* = 191)				ORO-PHARYNGEAL (*n* = 200)				URINE (*n* = 190)			
	Total (%)	HIV- (*n* = 106)	HIV+ (*n* = 85)	*P*-value	Total (%)	HIV- (*n* = 112)	HIV+ (*n* = 88)	*P*-value	Total (%)	HIV- (*n* = 107)	HIV+ (*n* = 83)	*P*-value
No type	52 (27.2)	45 (42.4)	7 (8.2)	˂0.001	177 (88.5)	104 (92.9)	73 (83.0)	0.029	161 (84.7)	91 (85.1)	70 (84.3)	0.892
Any type	139 (72.8)	61 (57.6)	78 (91.8)	˂0.001	23 (11.5)	8 (7.1)	15 (17.1)	0.029	29 (15.3)	16 (14.3)	13 (14.8)	0.892
Single type	26 (13.6)	21 (19.8)	5 (5.9)	0.005	16 (8.0)	6 (5.4)	10 (11.4)	0.120	22 (11.6)	14 (12.5)	8 (9.1)	0.461
Multiple types	113 (59.2)	40 (37.7)	73 (85.9)	˂0.001	7 (3.5)	2 (1.8)	5 (5.7)	0.136	7 (3.7)	2 (1.8)	5 (5.7)	0.131
LR-HPV	126 (66.0)	50 (47.2)	76 (89.4)	˂0.001	14 (7.0)	4 (3.6)	10 (11.4)	0.032	19 (10.0)	8 (7.5)	11 (13.3)	0.188
HR-HPV	110 (57.6)	41 (38.7)	69 (81.2)	˂0.001	15 (7.5)	6 (5.4)	9 (10.2)	0.194	15 (7.9)	9 (8.4)	6 (7.2)	0.764
HPV-6/11	50 (26.2)	19 (17.9)	31 (36.5)	0.003	3 (1.5)	1 (0.9)	2 (2.3)	0.425	3 (1.6)	0 (0.0)	3 (3.6)	0.047
HPV-16/18	55 (28.8)	19 (17.9)	36 (42.4)	˂0.001	6 (3.0)	3 (2.7)	3 (3.4)	0.763	3 (1.6)	2 (1.9)	1 (1.2)	0.715
HPV-6/11/16/18	77 (40.3)	30 (28.3)	47 (55.3)	˂0.001	9 (4.5)	4 (3.6)	5 (5.7)	0.474	6 (3.2)	2 (1.9)	4 (4.8)	0.248
HPV-31/33/45	50 (26.2)	14 (13.2)	36 (42.4)	˂0.001	3 (1.5)	0 (0.0)	3 (3.4)	0.048	5 (2.6)	3 (2.8)	2 (2.4)	0.866
HPV-6/11/16/18/31/33/45	98 (51.3)	36 (34.0)	62 (72.9)	˂0.001	11 (5.5)	4 (3.6)	7 (8.0)	0.177	10 (5.3)	5 (4.7)	5 (6.0)	0.679

**Table 3:** HPV infection among MSM only and MSMW at 3 anatomical sites among MSM in Cape Town, South Africa

HPV type classification	ANAL (*n* = 191)				ORO-PHARYNGEAL (*n* = 200)				URINE (*n* = 190)			
	Total (%)	MSM only (*n* = 150)	MSMW (*n* = 41)	*P*-value	Total (%)	MSM only (*n* = 155)	MSMW (*n* = 45)	*P*-value	Total (%)	MSM only (*n* = 145)	MSMW (*n* = 45)	*P*-value
No type	52 (27.2)	22 (14.8)	30 (73.2)	<0.001	177 (88.5)	133 (85.8)	44 (97.8)	0.027	161 (84.7)	123 (84.8)	38 (84.4)	0.950
Any type	139 (72.8)	128 (85.3)	11 (26.8)	<0.001	23 (11.5)	22 (14.2)	1 (2.2)	0.027	29 (15.3)	22 (15.2)	7 (15.6)	0.950
Single type	26 (13.6)	22 (14.7)	4 (9.8)	0.416	16 (8.0)	15 (9.7)	1 (2.2)	0.105	22 (11.6)	18 (12.4)	4 (8.9)	0.519
Multiple types	113 (59.2)	106 (70.7)	7 (17.1)	<0.001	7 (3.5)	7 (4.5)	0 (0.0)	0.147	7 (3.7)	4 (2.8)	3 (6.7)	0.224
LR-HPV	126 (66.0)	117 (78.0)	9 (22.0)	<0.001	14 (7.0)	13 (8.4)	1 (2.2)	0.154	19 (10.0)	15 (10.3)	4 (8.9)	0.776
HR-HPV	110 (57.6)	102 (68.0)	8 (19.50	<0.001	15 (7.5)	15 (9.7)	0 (0.0)	0.030	15 (7.9)	10 (6.9)	5 (11.1)	0.359
HPV-6/11	50 (26.2)	48 (32.0)	2 (4.9)	<0.001	3 (1.5)	3 (1.9)	0 (0.0)	0.347	3 (1.6)	3 (2.1)	0 (0.0)	0.331
HPV-16/18	55 (28.8)	52 (34.7)	3 (7.3)	<0.001	6 (3.0)	6 (3.9)	0 (0.0)	0.180	3 (1.6)	2 (1.4)	1 (2.2)	0.692
HPV-6/11/16/18	77 (40.3)	73 (48.7)	4 (9.8)	<0.001	9 (4.5)	9 (5.8)	0 (0.0)	0.098	6 (3.2)	5 (3.5)	1 (2.2)	0.681
HPV-31/33/45	50 (26.2)	48 (32.0)	2 (4.9)	<0.001	3 (1.5)	3 (1.9)	0 (0.0)	0.347	5 (2.6)	2 (1.4)	3 (6.7)	0.053
HPV-6/11/16/18/31/33/45	98 (51.3)	94 (62.7)	4 (9.8)	<0.001	11 (5.5)	11 (7.1)	0 (0.0)	0.066	10 (5.3)	6 (4.1)	4 (8.9)	0.212

For anal specimens, a total of 110 (57.6 %) [95 % CI: 50.3–64.7 %] were HR-HPV positive. HR-HPV types were less prevalent in the pharynx and urine, with 15 (7.5 %) [95 % CI: 4.3–12.1 %] oro-pharyngeal specimens and 15 (7.9 %) [95 % CI: 4.5–12.7 %] urine specimens testing positive for HR-HPV. HPV types 16 and 18, the only HR-HPV types included in both the bivalent and quadrivalent HPV vaccines, were detected in 55 (28.8 %) [95 % CI: 22.5–35.8 %] anal specimens, 6 (3.0 %) [95 % CI: 1.1–6.4 %] oro-pharyngeal specimens and 3 (1.6 %) [95 % CI: 0.3–4.5 %] urine specimens. The prevalence rates of all HPV genotypes included in the quadrivalent HPV vaccine (HPV-6/11/16/18) were 77 (40.3 %) [95 % CI: 33.3–47.6 %], 9 (4.5 %) [95 % CI: 2.1–8.4 %] and 6 (3.2 %) [95 % CI: 1.2–6.8 %] in anal, oro-pharyngeal and urine samples, respectively. HPV types included in the new 9-valent HPV vaccine (HPV-6/11/16/18/31/33/45/52/58) were detected in 110 (57.6 %) [95 % CI: 50.3–64.7 %], 12 (6.0 %) [95 % CI: 3.1–10.3 %] and 10 (5.3 %) [95%CI: 2.6–9.5 %] anal, oro-pharyngeal and urine specimens, respectively.

**Fig. 2:**
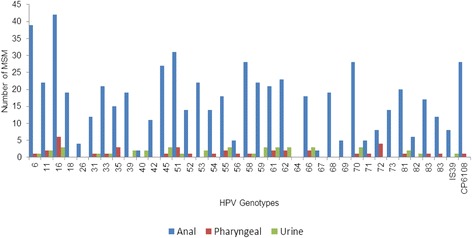
HPV type-specific prevalence rates in anal, oro-pharyngeal and urine specimens obtained from MSM participants in Cape Town

The most common HPV types detected in anal specimens were HPV-16 (42; 22 %), HPV-6 (39; 20.4 %) and HPV-51 (31; 16.2 %). In oro-pharyngeal specimens, the most common HPV types detected were HPV-16 (6; 3.0 %), HPV-72 (4; 2.0 %), followed by HPV-35 and HPV-51 (both 3; 1.5 %). The most prevalent HPV types in urine were HPV-16, HPV-45, HPV-55, HPV-59, HPV-61, HPV-62, HPV-66 and HPV-70 (all 3; 1.6 %). The HPV type specific prevalence rates in anal, oro-pharyngeal and urine specimens are summarised in Fig. 2.

### HPV and HIV co-infections

A total of 88 (44.0 %) participants tested HIV-positive in this study. Among the 85 HIV-positive participants with valid anal HPV results, 78 (91.8 %) were co-infected with any anal HPV (*p* < 0.001), 73 (85.9 %) had multiple anal HPV types (*p* < 0.001), 76 (89.4 %) had LR-HPV (*p* < 0.001) and 69 (81.2 %) were co-infected with HR-HPV (*p* < 0.001) (Table 2). Quadrivalent HPV vaccine types 6, 11, 16 and 18 were detected in 47 (55.3 %) anal specimens of HIV-positive participants (*p* < 0.001), and increased to 62 (72.9 %) with the addition of cross-protection HPV types 31, 33 and 45 (*p* < 0.001). Having any oro-pharyngeal HPV infection (15/88; 17.1 %) and having a LR-HPV oro-pharyngeal infection (10/88; 11.4 %) were significantly associated with being HIV-positive (*p* = 0.029 and *p* = 0.032 respectively). Multiple anal HPV types were detected more frequently in HIV-positive MSM (85.9 % vs 37.7 % in HIV-negative MSM). The mean number of HPV types detected in HPV-positive anal samples was 5.7 (range 1–15) in HIV-positive participants and 2.9 (range 1–10) in HIV-negative participants (*p* < 0.001).

### HPV according to sex partner status

Significant differences in anal HPV prevalence were observed between men who had sex with men only (MSM only) and men who had sex with men and women (MSMW) (Table 3). MSMW were less likely to have any anal HPV (85.3 % vs.26.8 %; *p* < 0.001), multiple anal HPV (70.7 % vs. 17.1 %; *p* < 0.001), anal LR-HPV (78.0 % vs. 22.0 %; *p* < 0.001), anal HR-HPV (68.0 % vs. 19.5 %; *p* < 0.001), anal HPV-6/11/16/18 (48.7 % vs. 9.8 %; *p* < 0.001) and anal HPV-6/11/16/18/31/33/45 (62.7 % vs. 9.8 %; *p* < 0.001). Individuals who had sex with men only were more likely to have any oro-pharyngeal HPV infection (14.2 % vs. 2.2 %; *p* = 0.027) and to have an oro-pharyngeal HR-HPV infection (9.7 % vs 0.0 %; *p* = 0.030). No significant differences were observed between MSM only and MSMW with regards to the prevalence of HPV in urine specimens.

### Risk factors associated with HPV infections

Univariate and multiple logistic regression analyses were performed to assess potential and independent associations between participant characteristics/sexual risk factors and HPV infection in this population (Table 4). In univariate analysis having any anal HPV infection was significantly associated with receiving an income (*p* = 0.044), having sex with men only (*p* < 0.001), engaging in group sex in their lifetime (*p* = 0.002), being HIV positive (*p* < 0.001) and practising receptive anal sex (*p* < 0.001). Having a HR-HPV infection in the anal canal was significantly associated with living outside the city (*p* = 0.050), transgender identity (*p* = 0.031), having sex with men only (*p* < 0.001), engaging in group sex in their lifetime (*p* = 0.006), sharing sex toys in last year (*p* = 0.042), being HIV positive (*p* < 0.001), practising receptive anal sex (*p* < 0.001) and having a gonococcal co-infection (*p* = 0.043). Multiple anal HPV infections were detected more frequently in participants that identified as transgender (*p* = 0.022), having sex with men only (*p* < 0.001), being HIV positive (*p* < 0.001), practising receptive anal sex (*p* < 0.001) and having a gonococcal co-infection (*p* = 0.028). Compared to other ethnicities, coloured MSM had significantly less anal HPV infections overall (*p* = 0.023), including HR-HPV (*p* = 0.003) and multiple HPV types (*p* = 0.013). Individuals who participated in transactional sex had fewer multiple anal HPV infections (*p* = 0.010). In multiple logistic regression analyses, having sex with men only (AOR: 10.9, 95 % CI: 3.88–30.81, *p* < 0.001), engaging in group sex in lifetime (AOR: 4.7, 95 % CI: 1.79–12.43, *p* = 0.002), being HIV-positive (AOR: 4.1, 95 % CI: 1.52–11.22, *p =* 0.006) and practising receptive anal intercourse (AOR: 3.3, 95 % CI: 1.36–8.09, *p =* 0.009) were the only factors associated with having any anal HPV infection after adjusting for confounders and other variables found significant in the univariate analysis. Coloured ethnicity was associated with significantly less anal HR-HPV infections (AOR: 0.4, 95 % CI: 0.13–0.96, *p =* 0.042) while having sex with men only (AOR: 4.6, 95 % CI: 1.63–12.74, *p =* 0.005), engaging in group sex in lifetime (AOR: 3.1, 95 % CI: 1.35–7.07, *p =* 0.006), being HIV-positive (AOR: 4.1, 95 % CI: 1.89–8.83, *p* < 0.001) and practicing receptive anal intercourse (AOR: 4.3, 95 % CI: 1.80–10.13, *p* < 0.001) were all associated with having a greater likelihood of infection by a HR-HPV type. Having multiple anal HPV infections were independently associated with having sex with men only (AOR: 5.5, 95 % CI: 1.92–15.51, *p =* 0.001), being HIV-positive (AOR: 6.9, 95 % CI: 3.10–15.42, *p* < 0.001) and practising receptive anal intercourse (AOR: 4.2, 95 % CI: 1.79–9.65, *p =* 0.001). Age group, education, job status, partner status, number of male sexual partners in the past 12 months, sex at sex-on-site venues, sex with a partner met through the internet, engaging in group sex in the past 12 months, alcohol and drug use during sex, having had a STI in the past 12 months, syphilis serostatus and having an anal chlamydial infection were not significantly associated with the three HPV outcomes in the anal canal. Further, no risk factors were independently associated with oro-pharyngeal HPV infections or with HPV detected in the urine.

**Table 4:** Univariate and multivariate logistic regression analyses for factors associated with any-, high-risk and multiple anal HPV infections

Risk factor	Any HPV					HR-HPV					Multiple HPV				
	No (%)	COR (95 % CI)	*p*	AOR (95 % CI)	*p*	No (%)	COR (95 % CI)	*p*	AOR (95 % CI)	*p*	No (%)	COR (95 % CI)	*p*	AOR (95 % CI)	*p*
Gender															
Male	124 (89.2)	Ref				97 (88.2)	Ref				99 (87.6)	Ref			
Transgender (MTF)	15 (10.8)	1.0				13 (11.8)	5.3 (1.16–24.16)	0.031			14 (12.4)	10.9 (1.40–84.63)	0.022		
Ethnicity															
Black	46 (33.1)	Ref		Ref		40 (36.4)	Ref		Ref		39 (34.5)	Ref			
Coloured	35 (25.2)	0.4 (0.18–0.88)	0.023	0.5 (0.15–1.42)	0.177	24 (21.8)	0.3 (0.15–0.67)	0.003	0.4 (0.13–0.96)	0.042	26 (23.0)	0.4 (0.19–0.82)	0.013		
White	55 (39.6)	1.2 (0.50–2.83)	0.685	0.5 (0.17–1.74)	0.303	45 (40.9)	0.9 (0.44–1.95)	0.847	0.5 (0.19–1.46)	0.219	47 (41.6)	1.2 (0.54–2.42)	0.717		
Other	3 (2.2)	0.9 (0.08–8.85)	0.890	0.2 (0.01–2.14)	0.162	1 (0.9)	0.2 (0.02–1.62)	0.121	0.0 (0.00–0.55)	0.016	1 (0.9)	0.2 (0.02–1.75)	0.137		
Residence															
City/suburbs	96 (69.1)	Ref				73 (66.4)	Ref				76 (67.3)	Ref			
Informal settlement	33 (23.7)	1.6 (0.72–3.73)	0.236			28 (25.5)	1.8 (0.88–3.73)	0.108			28 (24.8)	1.7 (0.80–3.42)	0.171		
Outside city	10 (7.2)	1.0				9 (8.2)	8.1 (1.00–65.96)	0.050			9 (8.0)	7.5 (0.92–60.49)	0.060		
Income status															
No income	24 (17.3)	Ref				18 (16.4)	Ref				20 (17.7)	Ref			
Income	115 (82.7)	2.1 (1.02–4.44)	0.044			92 (83.6)	1.9 (0.94–3.85)	0.072			93 (82.3)	1.6 (0.80–3.23)	0.187		
Sexual behaviour															
Sex partners															
Men and women	11 (2.9)	Ref		Ref		8 (7.3)	Ref		Ref		7 (6.2)	Ref		Ref	
Men only	128 (92.1)	15.9 (6.95–36.24)	<0.001	10.9 (3.88–30.81)	<0.001	102 (92.7)	8.8 (3.75–20.41)	<0.001	4.6 (1.63–12.74)	0.005	106 (93.8)	11.7 (4.82–28.39)	<0.001	5.5 (1.92–15.51)	0.001
Engaged in group sex in lifetime															
No	40 (28.8)	Ref		Ref		30 (27.3)	Ref		Ref		34 (30.1)	Ref			
Yes	99 (71.2)	2.9 (1.50–5.57)	0.002	4.7 (1.79–12.43)	0.002	80 (72.7)	2.4 (1.29–4.32)	0.006	3.1 (1.35–7.07)	0.006	79 (69.9)	1.8 (0.98–3.28)	0.057		
Shared sex toys in past 12 months															
No	111 (79.9)	Ref				85 (77.3)	Ref				88 (77.9)	Ref			
Yes	28 (20.1)	1.9 (0.75–4.98)	0.172			25 (22.7)	2.4 (1.03–5.36)	0.042			25 (22.1)	2.2 (0.95–4.97)	0.064		
Transactional sex															
No	92 (66.2)	Ref				74 (67.3)	Ref				79 (69.9)	Ref			
Paid and/or received money	47 (33.8)	0.6 (0.29–1.05)	0.072			36 (32.7)	0.6 (0.34–1.10)	0.100			34 (30.1)	0.5 (0.25–0.82)	0.010		
HIV serostatus															
Negative	61 (43.9)	Ref		Ref		41 (37.3)	Ref		Ref		40 (35.4)	Ref		Ref	
Positive	78 (56.1)	8.2 (3.46–19.50)	<0.001	4.1 (1.52–11.22)	0.006	69 (62.7)	6.8 (3.50–13.36)	<0.001	4.0 (1.82–8.65)	<0.001	73 (64.6)	10.0 (4.86–20.74)	<0.001	6.9 (3.10–15.42)	<0.001
Receptive anal intercourse with men															
Never practised	24 (17.3)	Ref				14 (12.7)	Ref		Ref		14 (12.4)	Ref		Ref	
Practised	115 (82.7)	7.7 (3.77–15.61)	<0.001	3.3 (1.36–8.09)	0.009	96 (87.3)	11.0 (4.97–24.21)	<0.001	4.3 (1.80–10.13)	0.001	99 (87.6)	11.3 (5.13–24.95)	<0.001	4.2 (1.79–9.65)	0.001
Anal gonorrhoea															
Negative	125 (89.9)	Ref				98 (89.1)	Ref				100 (88.5)	Ref			
Positive	14 (10.1)	1.0				12 (10.9)	4.8 (1.05–22.25)	0.043			13 (11.5)	10.0 (1.28–78.19)	0.028		

## Discussion

This study reports HPV prevalence and risk factors associated with HPV infections at three different anatomical sites among high-risk MSM in Cape Town. We found a high prevalence of HR-HPV (57.6 %) in the anal canal of study participants. HPV genotypes were less frequently detected in the pharynx (11.5 %) and urine (15.3 %) of study participants. Previous studies have shown that anal HPV infection is more common among MSM than heterosexual men (MSW), and that anal HPV prevalence rates can be 4–10 times higher in MSM compared to MSW [19, 20]. The high prevalence of anal HPV infection observed in this study is similar to anal HPV prevalence rates for high-risk MSM reported in Peru, Italy and Thailand but higher than anal HPV prevalence rates reported in countries such as Australia and Puerto Rico [9, 21–24].

MSM who are HIV-positive have an increased risk of anal HPV infection and anal cancer. HPV prevalence rates of 89–93 % have been reported among HIV-positive MSM in a recent systematic review and meta-analysis of 53 studies [7]. The incidence of anal cancer among HIV-positive MSM is more than 80-fold higher than that observed in the general population [25]. HIV infection has been shown to increase susceptibility to persistent HPV, increasing the risk of acquiring new HPV infections and reactivation of latent HPV infections [12]. Persons who are at high risk for HIV acquisition may be at higher risk of HPV infection due to the same high risk sexual practises [11]. As shown in Table 2, HIV-positive MSM had significantly more anal HPV infections overall (91.8 % HIV-infected vs. 57.6 % HIV-uninfected), including LR-HPV, HR-HPV, multiple HPV types and HPV vaccine types (HPV-6/11/16/18 + 31/33/45). The most common LR-HPV and HR-HPV types detected in the anal specimens of all MSM as well as HIV-positive MSM were HPV-6 and HPV-16 respectively, followed by high risk types HPV-58, HPV-45 and HPV-51. HPV-16 was also the most common HR-HPV type found in HIV-positive MSM from Thailand, USA, China, Spain and Canada [9, 12, 26–28].

Multiple anal HPV infections are commonly described among MSM, especially HIV-positive MSM [8, 26–28]. Having more than one HPV type detected was more common in the anal canal of MSM while single HPV infections were more frequently detected in the pharynx and urine. In this study, multiple HPV types were detected in the anal specimens of 59.2 % of all MSM, 81.3 % of all HPV-positive MSM and 85.9 % of HIV-positive MSM. Among an HIV-positive MSM cohort in Canada (HIPVIRG cohort study), the majority of participants (90.9 %) were infected with multiple HPV types in the anal canal, ranging between 0 and 18 HPV types (mean 5 HPV types) [12]. More recently, Supindham et al*.* [9] reported a 100 % HPV co-infection rate in the anal specimens of HIV-positive MSM in Thailand, with the majority being multiple HPV infections (mean 4.8 HPV types). The mean number of anal HPV types detected in HPV-positive MSM in this study was 4.5 types, which increased to 5.7 types among HIV-positive MSM (*p* < 0.001). The highest number of HPV types detected in a single anal sample was 15 HPV types in an HIV-positive participant co-infected with anal gonorrhoea and chlamydia. The mean number of HPV types in the oropharynx and urine was 1.7 and 1.4 types respectively, and the number of HPV types at these anatomical sites did not differ significantly according to HIV status. Multiple anal HPV infections are a cause for concern due to the strong association with anal intraepithelial neoplasia (AIN) and the development to high-grade AIN and anal cancer [29, 30].

Oro-pharyngeal HPV infections are much less common in adults than anogenital HPV infections [31]. King et al. [14] reported that the majority of HPV infections detected in the oral cavity of MSM in London were single HPV types. However, the incidence of HPV-related oro-pharyngeal cancers in the general population is increasing relative to other oral cancers associated with alcohol and tobacco use. A number of HR-HPV types have been implicated in carcinogenesis and tumour progression at different anatomical sites and are mainly due to the actions of their E6 and E7 viral oncogenes, which induce and maintain cellular transformation [32]. The majority of oro-pharyngeal squamous cell carcinomas (OPSCC) (+ − 90 % of cases) are associated with HPV-16 infection; however other HR-HPV types have also been identified in HPV-positive OPSCC and these types, although rare, constitute an important subset of oro-pharyngeal cancers which should not be ignored [33]. A few studies have reported associations between non-HPV-16 high-risk types and oro-pharyngeal cancer after controlling for or excluding HPV-16 infection [34–36]. A large US case–control study demonstrated that combined infections with HPV-16 and other HR-HPV types significantly increased the risk of head and neck squamous cell carcinoma [36]. Further studies are needed to determine whether oro-pharyngeal tumours containing non-HPV-16 high-risk types present less often with lymph node metastases and have better treatment outcomes [33, 37]. Davidson et al [38] reported a HPV prevalence of 5.6 % in oral rinse and gargle samples among male factory workers in Pretoria, South Africa, aged 17 - 64 years. Although oral sex was not common in this population, it was practised among men identified with HR-HPV infection [38]. Performing oral and oro-anal sex on men and women were not associated with oro-pharyngeal HPV infections in this study. However, HIV-positive MSM had significantly more oro-pharyngeal HPV infections (17.1 % in HIV-positive vs. 7.1 % in HIV-negative; *p* = 0.029), including LR-HPV infections (11.4 % in HIV-positive vs. 3.6 % in HIV-negative; *p* = 0.032). It is not clear if the increased oral HPV incidence in HIV-positive individuals is due to HIV-related immunosuppression or due to risky sexual practises [39]. In Italy, Parisi et al*.* [40] detected HPV in the oral specimens of approximately 20 % of HIV-infected MSM, which is lower than that reported by Blas et al*.* [41] (44.3 %) in Peru, but higher than the oro-pharyngeal HPV prevalence rates in HIV-positive MSM reported in this study. In our study, having any HPV infection or a LR-HPV infection in the oropharynx were the only oro-pharyngeal HPV groupings associated with being HIV-positive. No differences were observed between HIV-negative and HIV-positive MSM with regards to the prevalence of single HPV types, multiple HPV types, HR-HPV or HPV vaccine types detected in the oropharynx.

This study showed that MSM only had over three times more anal HPV infections and over 6 times more oro-pharyngeal HPV infections than MSMW. Anal HPV prevalence rates among MSM only were significantly higher for all HPV groupings, except for single HPV type prevalence. Having any oro-pharyngeal HPV infection or an oro-pharyngeal HR-HPV infection were the only oro-pharyngeal HPV groupings that showed significant differences between MSM only and MSMW. No significant differences were observed in the prevalence of HPV in urine between MSM only and MSMW. Compared to other male populations higher anal HPV prevalence rates are frequently reported for MSM only [19, 24, 42]. Limited information is available in the literature describing HPV prevalence rates in MSMW, especially anal HPV prevalence rates. Lu et al*.* [43] studied the seroprevalence of quadrivalent HPV vaccine types (HPV-6,11,16,18) among men with different sexual practises in Brazil, Mexico and USA (The *HIM* study) and found MSMW to have lower HPV seroprevalence rates than MSM but higher than in MSW. Genital HPV prevalence analysis in the same *HIM* study cohort showed that MSMW had higher HPV prevalence rates on the penis and scrotum compared to MSM and MSW, which could be attributed to increased number of sex partners and increased frequency of insertive sex acts among MSMW. In our study, in comparison with the MSM only group, significantly fewer MSMW were HIV-positive, practised receptive anal intercourse with men whilst more MSMW practised insertive anal intercourse with men. These sexual practises could account for the absence of gonococcal and chlamydial co-infections detected in the anal canal of MSMW and the increased number of chlamydial co-infections detected in the urine of MSMW. MSMW were also less likely to be syphilis seropositive. Even though anal HPV infection among MSMW who practise receptive anal intercourse could be linked to these sexual practises, anal HPV transmission can also be attributed to other factors, similar to those found among HPV-infected MSW, such as transmission through skin contact, sharing sex toys or vertical transmission. Genital HPV genotypes have been detected on fingers and HPV transmission between anogenital sites and hands have previously been reported among heterosexual couples [44]. These alternative modes of HPV transmission could explain the observation that 17.3 % of men who indicated no history of receptive anal intercourse had HPV detected in the anal canal.

This study had a number of limitations. Firstly, the relatively small sample size may have resulted in the misidentification of some predictors of HPV infection, especially in oro-pharyngeal and urine samples where the prevalence of HPV was low. Secondly, the liquid oral rinse sampling method has been shown to have a higher sensitivity than oro-pharyngeal swabs for the detection of HPV in the mouth and throat and therefore the use of oro-pharyngeal swabs in this study could have decreased the HPV detection rate in the oropharynx [13]. Thirdly, we were only able to test DNA extracts from urine as an indicator of urethral HPV infection. This was due to the sampling undertaken in the primary study; the true prevalence of penile HPV may have been higher than that reported here if penile samples had also been available for additional HPV testing. Fourthly, the participants attended one clinic in Cape Town and the findings of this study might not be generalized to the South African MSM population.

In keeping with South Africa’s National Strategic Plan for HIV and AIDS, STIs and TB, scaling up services for most at risk populations (including MSM) to reduce HIV/STI burden should be prioritised. For HPV, these efforts include targeted behavioural interventions such as promotion of proper and consistent condom use, medical male circumcision to reduce the incidence, prevalence and persistence of HPV infections in MSM and HPV vaccination of boys. The South African government is currently providing free HPV vaccinations for 9–10 year old girls in all public schools in South Africa but it is unlikely that MSM would benefit from herd immunity due to female vaccination roll-out and it would be optimal to vaccinate boys directly. Randomised controlled trials have shown HPV vaccination to be highly efficacious in MSM with the potential to substantially reduce the burden of HPV-associated disease in this population [45]. More studies are needed to determine the natural history and burden of HPV-associated diseases in South African MSM in order to formulate effective HPV prevention strategies for the future.

## Conclusions

A high prevalence of anal HPV infection was observed in this MSM population in Cape Town. All HPV groupings (any HPV, LR-HPV, HR-HPV, single type, multiple type, quadrivalent vaccine types) were more prevalent in the anal canal of HIV-positive MSM. Risk factors associated with any HPV, HR-HPV and multiple HPV types in the anal canal included having sex with men only, being HIV-positive and practising receptive anal intercourse. No risk factors were independently associated with oro-pharyngeal HPV infection or HPV detected in urine. Targeted interventions and HPV vaccination of boys should be considered to reduce the burden of HPV-related diseases among MSM in South Africa.

## Abbreviations

AOR, adjusted odds ratio; CI, confidence interval; COR, crude odds ratio; HIV, human immunodeficiency virus; HPV, human papillomavirus; HR, high-risk; IQR, interquartile range; ISH, *in situ* hybridisation; ITCMH, Ivan Toms Centre for Men’s Health; LA, linear Array; LR, low-risk; MSM, men-who-have-sex-with-men; MSMW, men-who-have-sex-with-men-and-women; MSW, men-who-have-sex-with-women; NICD, National Institute for Communicable Diseases; SD, standard deviation; SPM, sexual partner matrix; STI, sexually transmitted infection; TGW, transgender women
